# Durable response to sotorasib in *KRAS G12C*-mutant intrahepatic cholangiocarcinoma: a case report

**DOI:** 10.1093/oncolo/oyag260

**Published:** 2026-07-06

**Authors:** Chaoxing Liu, Kang Li, Jingjian Feng

**Affiliations:** Department of Radiation Oncology, Shijiazhuang People’s Hospital, Shijiazhuang, Hebei Province, China; Department of Radiation Oncology, Shijiazhuang People’s Hospital, Shijiazhuang, Hebei Province, China; Department of Radiation Oncology, Shijiazhuang People’s Hospital, Shijiazhuang, Hebei Province, China

**Keywords:** case report, KRAS G12C, sotorasib, intrahepatic cholangiocarcinoma, targeted therapy

## Abstract

KRAS G12C mutations are rare in intrahepatic cholangiocarcinoma (iCCA), occurring in approximately 1% of cases. While adagrasib has shown efficacy in biliary tract cancers in clinical trials, real-world evidence for sotorasib remains limited. We report a 64-year-old male with advanced iCCA harboring a *KRAS p.(G12C)* mutation (58.07% allelic frequency) who progressed on multiple prior therapies including chemotherapy, immunotherapy, and targeted therapy. Disease progression was complicated by obstructive jaundice and the Eastern Cooperative Oncology Group (ECOG) performance status deterioration to 3, requiring percutaneous transhepatic cholangiodrainage (PTCD). Following initiation of sotorasib 480 mg daily, the patient achieved a marked partial radiographic response within 6 weeks. CA19-9 normalized, jaundice resolved, PTCD was removed, and performance status improved to ECOG 1. The patient received continuous sotorasib for 10 months, with the initial partial response lasting 8 months with no significant treatment-related adverse events. This case provides real-world evidence that sotorasib can induce durable responses in chemotherapy-refractory, *KRAS G12C*-mutant iCCA, including in patients with poor performance status and obstructive jaundice who would typically be excluded from clinical trials.

**Key points** 

- A patient with chemotherapy-refractory, *KRAS G12C*-mutant intrahepatic cholangiocarcinoma (iCCA) experienced a durable partial response lasting 8 months on sotorasib, despite having poor performance status (ECOG 3) and obstructive jaundice requiring biliary drainage.
- This case provides real-world evidence that *KRAS G12C* inhibition can be effective in iCCA, a rare molecular subtype (1.1% of iCCA), and supports comprehensive genomic profiling to identify actionable drivers in advanced biliary tract cancers.

**Patient story** 

A 64-year-old man presented with incidental liver and lung masses. After progressing on multiple standard therapies, he developed severe obstructive jaundice, became bedridden (ECOG 3), and required a percutaneous biliary drainage tube. Genomic testing revealed a rare *KRAS G12C* mutation. He was started on sotorasib, a targeted therapy. Within weeks, his jaundice resolved, the drainage tube was removed, and he returned to normal activities. His cancer shrank and remained controlled for 8 months.

**Molecular tumor board** 

- Tumor characteristics: Advanced intrahepatic cholangiocarcinoma with *KRAS p.(G12C)* mutation (58.07% allelic frequency). No other actionable alterations detected (*TP53, PIK3CA, BRAF, NRAS, MET, FGFR2, IDH1/2* wild-type). Microsatellite stable.
- Treatment recommendation: Off-label sotorasib 480 mg daily, based on *KRAS G12C* inhibition rationale and absence of known resistance co-alterations.
- Clinical outcome: Partial response (RECIST) lasting 8 months; eventual progression at month 14.

**Patient update** 

As of the data cutoff (month 17), the patient received 10 months of continuous sotorasib. The initial partial response lasted 8 months. Progressive disease was confirmed at month 14. The patient remained on sotorasib until month 14, when therapy was discontinued due to overall progression. Supportive care was initiated and then the patient was lost to follow-up.

## Introduction

Intrahepatic cholangiocarcinoma (iCCA) is an aggressive hepatobiliary malignancy with rising global incidence and poor prognosis.^1^ The standard-of-care first-line therapy for advanced biliary tract cancers has recently evolved. Both the TOPAZ-1 trial (durvalumab plus gemcitabine and cisplatin) and the KEYNOTE-966 trial (pembrolizumab plus gemcitabine and cisplatin) have demonstrated superior overall survival compared with chemotherapy alone, establishing chemoimmunotherapy as the new standard.^2^^,^^3^ However, effective treatment options for patients who progress on or after frontline therapy remain limited and largely unstandardized.

The therapeutic landscape for iCCA has been transformed by the adoption of comprehensive genomic profiling, which has revealed a diverse array of actionable driver alterations, underscoring iCCA as a model disease for precision oncology. For patients with FGFR2 fusions or rearrangements, the selective *FGFR* inhibitors pemigatinib and futibatinib are approved based on the FIGHT-202 and FOENIX-CCA2 trials, respectively.^4^^,^^5^ Those harboring IDH1 mutations may benefit from ivosidenib, an *IDH1* inhibitor approved on the basis of the phase 3 ClarIDHy trial.^6^ Although rare, patients with microsatellite instability-high tumors are eligible for pembrolizumab irrespective of tumor type. More recently, the *HER2*-directed antibody-drug conjugate zanidatamab has shown promising activity in *HER2*-amplified Biliary Tract Cancer (BTC).^7^ Despite these advances, no targeted therapy has been approved for the 23% of iCCA patients harboring *KRAS* mutations, particularly the rare *KRAS G12C* variant, which accounts for only 1.1% of iCCA cases.^8^^,^^9^

Sotorasib, a first-in-class *KRAS G12C* inhibitor, is approved for *KRAS G12C*-mutant non-small cell lung cancer.^10^ However, published experience with sotorasib in iCCA remains limited to a single stable disease case in a basket trial. Real-world evidence is needed to validate its activity outside clinical trial settings, particularly in patients with poor performance status or complications such as obstructive jaundice who are typically excluded from trials.

## Case presentation

### Patient information and initial diagnosis

A 64-year-old asymptomatic male presented at month 0 (August 2022) with incidental liver and lung masses found on routine health examination. He had no history of viral hepatitis, chronic liver disease, or malignancy in first-degree relatives. The Eastern Cooperative Oncology Group (ECOG) performance status was 1.

Initial staging investigations included contrast-enhanced CT of the chest at month 0 (August 2022), revealing multiple bilateral pulmonary nodules. Abdominal MRI at month 0 demonstrated multiple intrahepatic masses consistent with metastatic disease. Tumor markers were markedly elevated: CA19-9 > 1000.00 U/mL, CA125 139.40 U/mL, and CEA 9.61 ng/mL.

^18^F-FDG PET/CT showed a hypermetabolic mass in the posterior segment of the right hepatic lobe with multiple intrahepatic hypermetabolic foci, consistent with iCCA with intrahepatic metastases. A liver biopsy confirmed poorly differentiated adenocarcinoma with immunohistochemistry positive for CK7, CK19, and CK8/18 and negative for hepatocellular markers. Ki-67 was approximately 20%.

Comprehensive genomic profiling was performed on the initial liver biopsy specimen using a validated next-generation sequencing panel (The Fourth Hospital of Hebei Medical University, in-house panel) encompassing 55 cancer-related genes, including *AKT1, ALK, BRAF, EGFR, ERBB2, FGFR1-4, IDH1/2, KRAS, MET, NRAS, PIK3CA, TP53, ROS1, RET*, and microsatellite instability sites. The assay was conducted on formalin-fixed paraffin-embedded tumor tissue with a tumor cellularity of 50%. The test identified a *KRAS p.(G12C)* missense mutation (*c.34G>T*) with an allelic frequency of 58.07%. No other clinically significant alterations were detected across the remaining 54 genes. Specifically, no pathogenic or likely pathogenic variants were identified in *TP53, PIK3CA, BRAF, NRAS, MET, FGFR2*, or *IDH1/2*. The tumor was determined to be microsatellite stable.

The patient’s case was formally presented and discussed at our institution’s Molecular Tumor Board (MTB), a multidisciplinary forum attended by medical oncologists, molecular pathologists, and clinical geneticists. The MTB reviewed the patient’s clinical history, prior therapies, and genomic findings. Based on the presence of the *KRAS G12C* mutation (58% allelic frequency), the absence of identified resistance co-alterations, and the lack of other actionable drivers, the MTB recommended off-label treatment with the *KRAS G12C* inhibitor sotorasib. Following this recommendation and after obtaining written informed consent from the patient for off-label use, the treating medical oncologist initiated sotorasib therapy.

### Prior treatments

The patient initially received two cycles of hepatic artery infusion chemotherapy (HAIC) at month 0 (September 2022) and month 1 (October 2022) with gemcitabine, oxaliplatin, and fluorouracil, complicated by grade 3 gastrointestinal toxicity and with progression on restaging MRI at month 2 (November 2022).

It is important to note that HAIC was selected because of the patient’s dominant, multifocal intrahepatic disease burden, with the goal of achieving maximal locoregional disease control. HAIC is an off-label, institutional practice and does not represent a guideline-recommended standard first-line therapy for advanced iCCA. Systemic therapy was then initiated with one cycle of nab-paclitaxel and cisplatin at month 2 (November 2022). Concurrently, the patient had been receiving lenvatinib (8 mg daily from month 0) and two cycles of durvalumab at months 1 and 2 (October and November 2022). This combination was based on exploratory data from the LEAP-005 basket trial suggesting potential activity in BTC; however, lenvatinib is not approved for biliary tract malignancies, and the combination with durvalumab is not a standard-of-care regimen. These earlier treatment choices are described for completeness but should not be interpreted as standards against which the efficacy of sotorasib should be compared.

### Progressive disease and targeted therapy

Despite these interventions, repeat MRI at month 3 (December 2022) confirmed ongoing progression. The patient developed obstructive jaundice (total bilirubin 12.4 mg/dL), ECOG declined to 3, and percutaneous transhepatic cholangiodrainage (PTCD) was placed at month 3 (December 2022).

At month 3 (December 2022), the patient initiated sotorasib 480 mg once daily. The 480 mg starting dose was selected by the treating oncologist based on the patient’s poor performance status (ECOG 3) and preexisting obstructive jaundice (total bilirubin 12.4 mg/dL), balancing the potential for antitumor activity with the risk of hepatotoxicity.

### Response and follow-up

- Month 5 (February 2023, 6 weeks on sotorasib): MRI showed marked reduction in intrahepatic tumor burden and improvement in biliary dilation. CA19-9 decreased from >1000 to 87 U/mL. Total bilirubin normalized. ECOG improved to 2.
- Month 6 (March 2023): Continued slight tumor regression.
- Month 8 (May 2023): Further response. ECOG 1, asymptomatic, CA19-9 normal, PTCD removed.
- Month 11 (August 2023): Ongoing response with no progression (8 months on sotorasib). The initial partial response was sustained for 8 months (from month 5 to month 13).
- Month 13 (October 2024): Progressive disease was confirmed by imaging.
- Month 17 (January 2025): Data cutoff; patient transitioned to supportive care and lost to follow-up.

### Safety

All adverse events (AEs) were graded according to CTCAE version 5.0. During the entire sotorasib treatment period, the patient experienced only grade 1 AEs, which were self-limited and did not require dose interruption or medical intervention. Specifically, grade 1 diarrhea occurred during the first month of therapy and resolved spontaneously without antidiarrheal medication. No grade ≥2 AEs were observed. Of particular note, no hepatotoxicity (grade ≥1 ALT, AST, or alkaline phosphatase elevation) occurred despite the patient’s baseline obstructive jaundice and preexisting hepatic dysfunction. No dose reductions, treatment interruptions, or hospitalizations were required for AE management.

## Discussion

**Figure 1.**
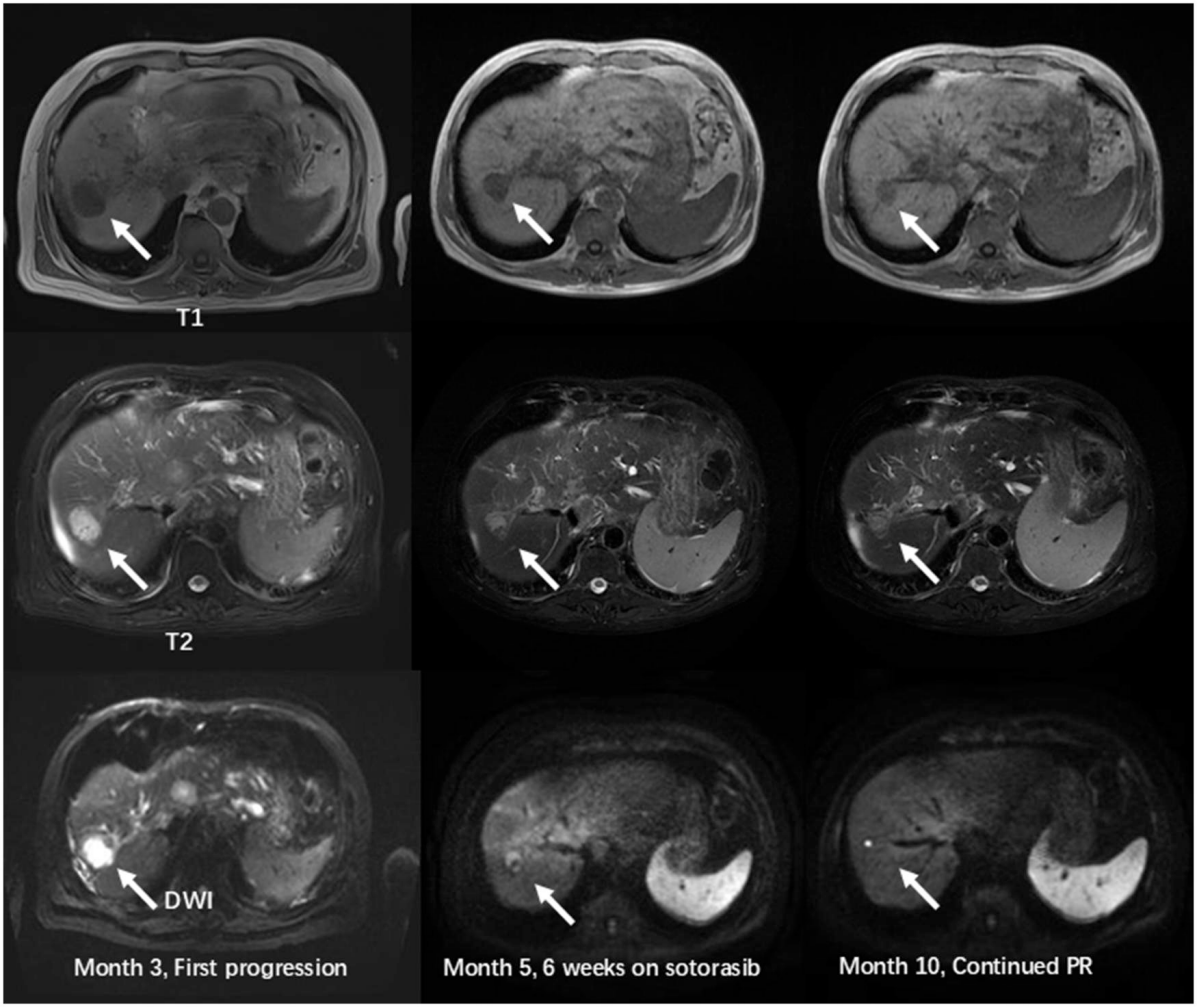
Three-panel T1-weighted magnetic resonance image of the liver. (A) At month 3, first progression showing multiple dark, solid metastatic lesions. (B) At month 5, 6 weeks after starting sotorasib showing those lesions have significantly shrunk and decreased in number. (C) At month 10 showing continued reduction, with only small residual lesions visible.

We report a patient with heavily pretreated, *KRAS G12C*-mutant iCCA who achieved a durable partial response lasting 8 months on sotorasib (Figure 1), despite having poor performance status (ECOG 3) and obstructive jaundice requiring PTCD.

First, this case provides real-world evidence for sotorasib activity in *KRAS G12C*-mutant cholangiocarcinoma. While the KRYSTAL-1 trial demonstrated efficacy of adagrasib in BTCs,^11^ data for sotorasib remain sparse. Our case confirms that sotorasib, a distinct *KRAS G12C* inhibitor, can produce similarly impressive responses in this rare molecular subset.

Second, our patient had features typically excluded from clinical trials (ECOG 3, obstructive jaundice requiring biliary drainage). This highlights an important gap between clinical trial populations and real-world patients. Our report demonstrates that sotorasib can be safely and effectively used even in such challenging scenarios.

**Figure 2.**
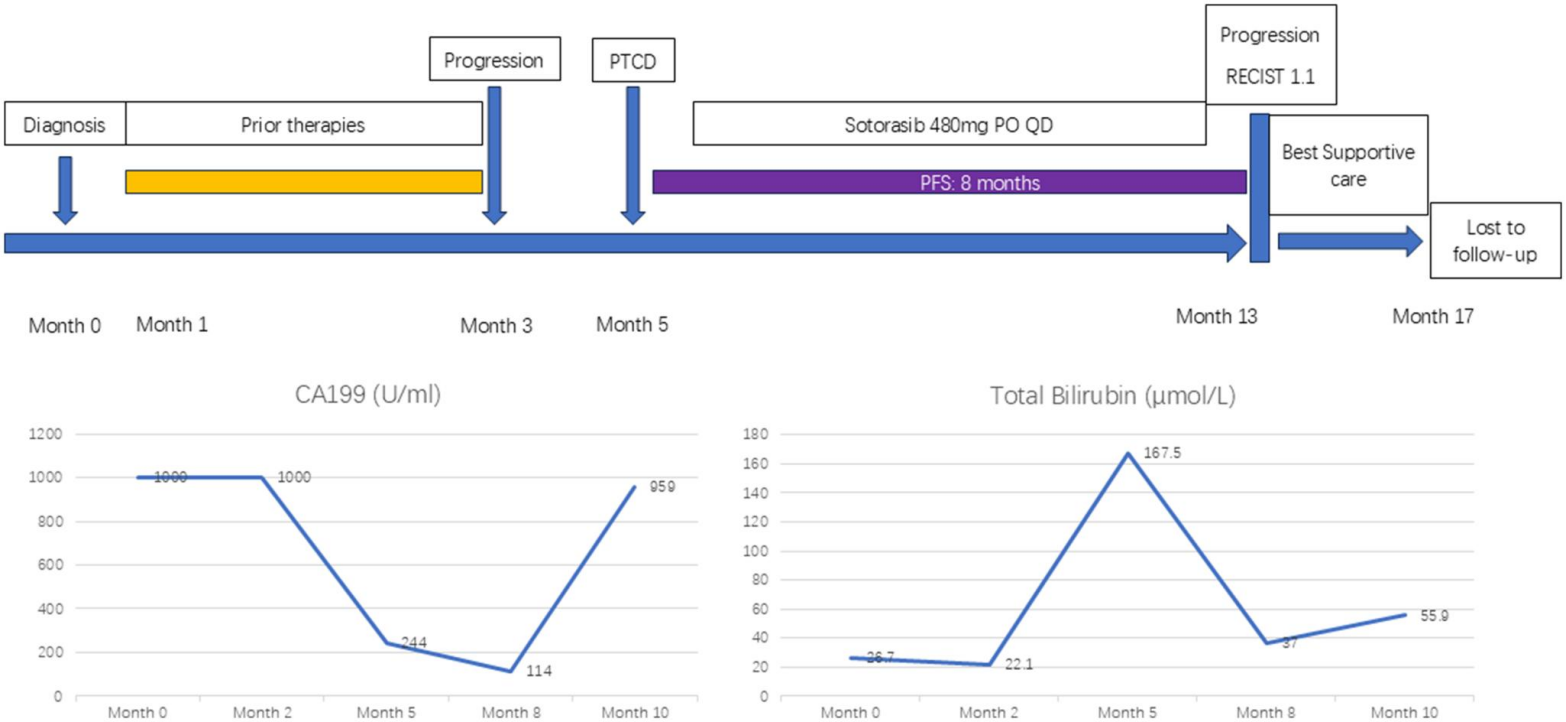
A swimmer’s plot shows the sequence of treatments (HAIC, nab-paclitaxel/cisplatin, lenvatinib/durvalumab, then sotorasib). A line graph below shows CA19-9 levels dropping from >1000 to 114 U/mL within 3 months and total bilirubin decreasing from 167.5 to 37.0 µmol/L within 2 months.

Third, the depth and durability of response (lasting 8 months) suggest that iCCA with high-allelic-frequency *KRAS G12C* mutations may be particularly sensitive to KRAS G12C inhibition. The rapid clinical improvement allowing PTCD removal and normalization of CA19-9 underscores the meaningful palliative benefit achievable with genotype-matched therapy. A late and transient elevation in CA19-9 observed at months 8 and 10 coincided with radiologic progression of a preexisting left portal vein tumor thrombus, whereas all other metastatic sites remained controlled or stable, suggesting that tumor marker fluctuations may be lesion specific and do not necessarily reflect global disease progression in the context of ongoing targeted therapy (Figure 2).

The efficacy observed in this patient must be contextualized within the broader clinical experience with *KRAS G12C* inhibitors in biliary tract cancers. To date, the most robust data come from the phase 1/2 KRYSTAL-1 trial of adagrasib, which reported a confirmed objective response rate of 41.7% (5/12 patients) and a disease control rate of 91.7% (11/12 patients) in the BTC cohort. The median duration of response was 5.3 months (95% CI, 2.8 to 7.3) and median progression-free survival (PFS) was 7.4 months (95% CI, 5.3 to 8.6). Treatment-related adverse events (TRAEs) of any grade were observed in 96.8% of patients and grade 3-4 in 27.0%; there were no grade 5 TRAEs.^11^ The 8-month initial response duration observed in our patient compares favorably with these published results. However, it is important to recognize that acquired resistance to *KRAS G12C* inhibitors is virtually universal with continued therapy. Preclinical and emerging clinical data have identified several resistance mechanisms, including on-target mutations within the KRAS G12C allele (e.g. secondary mutations at *Y96D, H95D/Q/R* that impair drug binding) and off-target pathway reactivation (e.g. *NRAS* or *BRAF* mutations, *MAP2K1* activating mutations, or upregulation of receptor tyrosine kinases such as *EGFR* or *MET*).^12^ Understanding these mechanisms will be essential for developing rational combination strategies and next-line therapies to extend the duration of clinical benefit in responding patients.

The 480 mg starting dose of sotorasib used in this patient requires justification, as the FDA-approved dose is 960 mg daily based on the CodeBreaK100 phase 2 trial.^13^^,^^14^ The decision to initiate sotorasib at 480 mg once daily was made by the treating oncologist after careful consideration of the patient’s pretreatment clinical status: an ECOG performance status of 3 and significant obstructive jaundice (total bilirubin 12.4 mg/dL) at the time of therapy initiation. Given the known association of higher sotorasib doses with hepatotoxicity, a reduced starting dose was selected to minimize the risk of further hepatic injury in a patient with already compromised liver function. Importantly, a growing body of evidence, including systematic reviews and meta-analyses, has since demonstrated comparable response rates between the approved 960 mg dose and reduced doses (pooled ORR 32% vs 26%; RR 1.26, 95% CI, 0.87-1.83), with no significant difference in PFS.^12^ This emerging evidence supports the clinical rationale for dose reduction in vulnerable populations and has informed ongoing FDA efforts under Project Optimus to identify the lowest effective dose for *KRAS G12C* inhibitors. The fact that our patient achieved a sustained partial response lasting 8 months on 480 mg daily further supports the potential efficacy of lower doses in selected patients.

We acknowledge limitations inherent to a single case report, including lack of generalizability. The optimal sequencing of *KRAS G12C* inhibitors relative to other therapies remains undefined.

In conclusion, this case suggests the potential for meaningful clinical activity with sotorasib in a heavily pretreated patient with *KRAS G12C*-mutant iCCA, even in the setting of poor performance status and malignant biliary obstruction. The outcomes we observed, together with published data on adagrasib in BTC, provide a rationale for considering *KRAS G12C* inhibitors in molecularly selected patients when standard options have been exhausted. Prospective clinical trials are needed to define the overall response rate, durability of benefit, and safety profile of sotorasib specifically in *KRAS G12C*-mutant biliary tract cancers.

## Data Availability

Data sharing is not applicable to this article as no datasets were generated or analyzed during the current study.
